# ELDER SELF-NEGLECT: REPORTING AND RESPONSE WITHOUT APS OVERSIGHT

**DOI:** 10.1093/geroni/igz038.1687

**Published:** 2019-11-08

**Authors:** Kelly A Melekis

**Affiliations:** Skidmore College, Saratoga Springs, New York, United States

## Abstract

Self-neglect is generally considered a geriatric phenomenon and subsumed within the category of elder abuse and neglect. In the U.S., self-neglect is the most commonly reported form of elder abuse and neglect and has been linked to decreased physical and psychological well-being, higher mortality rate, and increase health care services utilization. While self-neglect typically falls under the purview of a state’s adult protective service (APS) system, there are several states where this is not codified and/or practiced. This study examined the state of existing laws, policies and programs that address elder self-neglect in the U.S. in an effort to better understand how self-neglect reporting and response is handled, particularly in states where APS does not have oversight. A 50-state review of existing policies and laws addressing elder self-neglect was followed by key stakeholder interviews with those who oversee self-neglect reporting and response in states where APS does not have oversight. Findings indicate that a range of rationales explain variation in responsibility for self-neglect cases, including a lack of specificity in statutes, concerns that self-neglect does not fit into legal/criminal models due to the lack of perpetrator/victim relationship, and strong sentiments that those engaging in self-neglect need human services and support rather than investigatory or legal approaches. While the inclusion of self-neglect into the broad category of elder abuse and neglect is often perceived as vital for the provision of services, findings indicate it may exacerbate the complex challenges inherent in defining and conceptualizing both the term and its response.

